# *Helicobacter pylori* Infection Aggravates Dysbiosis of Gut Microbiome in Children With Gastritis

**DOI:** 10.3389/fcimb.2019.00375

**Published:** 2019-11-07

**Authors:** Lu Yang, Jiaming Zhang, Junjie Xu, Xuxia Wei, Junjie Yang, Yi Liu, Hua Li, Changying Zhao, Ying Wang, Lei Zhang, Zhongtao Gai

**Affiliations:** ^1^Department of Digestive Disease, Qilu Children's Hospital of Shandong University, Jinan, China; ^2^Shandong Children's Microbiome Center, Qilu Children's Hospital of Shandong University, Jinan, China; ^3^College of Life Science, Qilu Normal University, Jinan, China; ^4^Research Institute of Pediatrics, Qilu Children's Hospital of Shandong University, Jinan, China; ^5^Beijing Advanced Innovation Center for Big Data-Based Precision Medicine, Beihang University, Beijing, China

**Keywords:** gastritis, gut microbiome, *Helicobacter pylori*, children, infection

## Abstract

**Introduction:** *Helicobacter pylori* infection consistently leads to chronic and low degree of inflammatory response in gastric mucosa and is closely related with gastrointestinal and extra-gastric diseases. Effects of local microbiome in the stomach have been studied in adults and children with *H. pylori* infection. It is, however, not known whether the intestinal microbial community differs in children with varying *H. pylori* infection. The aim of this study is to characterize the altered composition of microbiome induced by *H. pylori* infection and in gastritis.

**Materials and Methods:** This study involved 154 individuals, including 50 children affected by *H. pylori*-induced gastritis, 42 children with *H. pylori*-negative gastritis, and 62 healthy controls. Gut microbiome composition was analyzed using 16S rRNA gene-based pyrosequencing. Fecal bacterial diversity and composition were then compared.

**Results:** On the basis of an analysis of similarities and differences, we found that children with *H. pylori*-induced gastritis exhibited gut bacteria dysbiosis. The ratio of Firmicutes/Bacteroidetes (F:B) at the phylum level had dramatically decreased in *H. pylori*-positive gastritis group (HPG) and *H. pylori*-negative gastritis group (HNG), compared with the healthy control group (HCG). At the family and genus levels, relative abundance of Bacteroidaceae and Enterobacteriaceae was prevalent in HPG and HNG, whereas relative abundance of Lachnospiraceae, Bifidobacteriaceae, and Lactobacillaceae was seen in HCG. Prevalence of different taxa of gut microbiome at the class, order, family, and genus levels was also observed among the three groups.

**Conclusions:** Gastritis can cause changes in composition of fecal microbiome, which is exacerbated by *H. pylori* infection. These changes in gut microbiome may be related to drug resistance and development of chronic gastrointestinal diseases.

## Introduction

*Helicobacter pylori* is a well-known pathogen in chronic gastritis, peptic ulcer, and gastric cancer (Marshall and Warren, 1984), usually acquired in early childhood, mostly before the age of 5 (Weyermann et al., 2009; O'Ryan et al., 2015). This infection causes a persistently chronic and low degree of inflammatory response in gastric and duodenal mucosa, which may persist lifelong without treatment. *H. pylori* infection is strain dependent and determined by its association with cytotoxic gene A (cag A) (Wang et al., 2016), bacterial characteristics, inflammatory response, host conditions, and environmental factors. China has a high prevalence of *H. pylori* infection. The infection rate is currently 40–60%, reaching as high as 37.1% before 20 years of infection stage. Early diagnosis and treatment can, therefore, prevent *H. pylori*-related complications (Sugano et al., 2015; Malfertheiner et al., 2017). The European and North American Societies for Pediatric Gastroenterology, Hepatology and Nutrition recommends the use of triple therapy, including proton pump inhibitors (PPIs) in combination with amoxicillin and either imidazole or clarithromycin, as the first line of treatment for *H. pylori* eradication in children (Koletzko et al., 2011). However, successful eradication is lower in children owing to poor drug compliance and antibiotic resistance displayed by *H. pylori* (Oderda et al., 2007; Okuda et al., 2017). Meta-analyses from previous studies have revealed that triple therapy supplemented with probiotics increased the eradication rate while decreasing the adverse reactions of triple therapy in *H. pylori* infection, especially in children (Szajewska et al., 2010; Li et al., 2014). An understanding of how the gastrointestinal microbiome interacts with *H. pylori* during infection might provide novel targets for its prevention and treatment in children.

Recent studies focusing on gastric microbiome showed that the interaction between *H. pylori* and other microbes may play a pivotal role in *H. pylori*-associated diseases (Bik et al., 2006; Schulz et al., 2015; Alarcon et al., 2017; Brawner et al., 2017; Llorca et al., 2017). Gastric microbiome is mainly represented by four phyla (Proteobacteria, Bacteroidetes, Firmicutes, and Actinobacteria) in the general population, and remarkable changes in gastric microbiome composition were observed between *H. pylori*-positive and *H. pylori*-negative individuals (Maldonado-Contreras et al., 2011; Alarcon et al., 2017). Positive *H. pylori* status was associated with increased abundance of proteobacteria, especially epsilonproteobacteria, mainly because of *Helicobacter* genus. An increased abundance of Spirochetes and Acidobacteria was also observed, with decreased abundance of Actinobacteria, Bacteroidetes, and Firmicutes (Maldonado-Contreras et al., 2011). Similar results were reported in *H. pylori*-infected children, presenting a higher relative abundance of *Helicobacter* genus (66.3%) than in *H. pylori*-negative children (0.45%) and lower bacterial diversity (Brawner et al., 2017; Llorca et al., 2017). Dysbiosis of gastric microbiota and some specific bacteria were found to be associated with gastric carcinoma or precancerous lesions (Coker et al., 2018; Ferreira et al., 2018). Notably, *H. pylori* played a crucial role in carcinogenesis. Some studies also suggested that the interaction between *Lactobacillus, Streptococcus*, and *H. pylori* enhances gastric inflammation and promotes *H. pylori*-associated carcinogenesis (Aviles-Jimenez et al., 2014; Rizzato et al., 2019).

For resident microorganisms, the host is a unique entity along the gastrointestinal tract, and any change in these factors would modify homeostasis. As mentioned above, the role of *H. pylori* infection on the gastric microbiome and the interaction in associated gastric diseases has been highlighted in recent studies. The interaction between *H. pylori* and gut microbiota has also been analyzed, although the exact underlying mechanism still remains unclear. Hypochlorhydria and hypergastrinemia caused by *H. pylori* infection were some of the causes in the interaction between *H. pylori* infection and gut microbiota (Beasley et al., 2015; He et al., 2016). Leptin and ghrelin secretion decreased in *H. pylori*-positive patients and indirectly also influenced the gastrointestinal microenvironment by modulating gastric acid secretion and immune response (La Cava and Matarese, 2004; Baatar et al., 2011; Francois et al., 2011; Muhsen et al., 2015; He et al., 2016). The plasma ghrelin level also significantly correlated with several kinds of bacteria, including *Bifidobacterium* and *Bacteroides*. Additionally, increased immune reaction caused by *H. pylori* infection is related to the genes in gastric and pulmonary tissues (Kienesberger et al., 2016). Animal studies have indicated that *H. pylori* results in distinct shifts in gut microbiota in distal, uninflamed parts of the gastrointestinal tract (Heimesaat et al., 2014). Similarly, several researches of human fecal samples on the relationship between *H. pylori* and intestinal flora also suggested a different composition of gut microbioma (Buhling et al., 2001; Myllyluoma et al., 2007; Chen et al., 2018; Gao et al., 2018; Iino et al., 2018). Some of the literatures we referred to focused on the changes of gut microbiome before and after treatment (Buhling et al., 2001; Myllyluoma et al., 2007; Chen et al., 2018). Myllyluoma et al. concluded that the concentration of clostridia and the total numbers of anaerobes significantly decreased in *H. pylori*-positive ones, compared with *H. pylori*-negative ones (Myllyluoma et al., 2007). Chen et al.'s study mainly focused on the impact of *H. pylori* eradication treatment using triple therapy. They also first compared the two groups on day 0 and observed significant increase in alpha diversity of *H. pylori*-positive fecal samples compared with *H. pylori*-negative subjects (Chen et al., 2018). Additionally, a Japanese study of 1,123 adult subjects, using 16S rRNA amplification from fecal samples, also confirmed higher abundance of *Lactobacillus* in *H. pylori*-infected subjects with severe atrophic gastritis (Iino et al., 2018). However, there has been only one study related to fecal microbiota and *H. pylori* infection in children, which included 18 fecal samples from five Japanese families (Osaki et al., 2018). These indicated a similar composition of intestinal microbiota between members of the same family, but the sample size was small. The relationship between *H. pylori*, gastritis, and gut microbiome variation has scarcely been analyzed (Chen et al., 2018; Gao et al., 2018), especially in children. In this study, fecal microbiome in children with *H. pylori*-positive gastritis group (HPG), *H. pylori*-negative gastritis group (HNG), and healthy control group (HCG) were compared before treatment using 16S rRNA gene sequence to confirm the impact of *H. pylori* infection and gastritis on gut microbiome. The results from this study may be useful in further evaluation of *H. pylori* infection, prevention, and treatment in children.

## Materials and Methods

### Study Design and Participants

**Figure 1:**
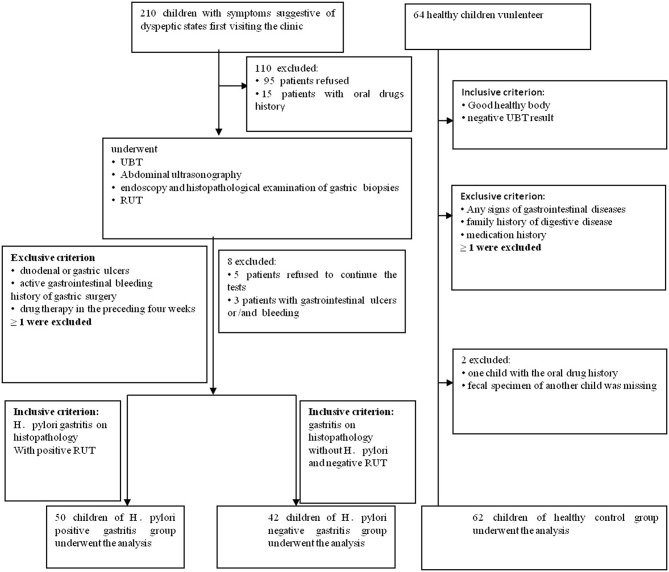
Flowchart of this study. 210 children with dyspeptic symptoms and 64 healthy children were initially screened for the study. Ninety-five individuals refused to donate fecal samples, and another 15 children had oral drug history. They all had been excluded. In the second part of the tests, five patients refused to continue all the tests, and another three patients with the gastrointestinal ulcers and/or bleeding were missed. In the healthy children group, one fecal specimen of the child was missing, and another child with oral drug history was ruled out.

This prospective pilot study, which was approved by the Institutional Review Boards at Qilu Children's Hospital of Shandong University (IRB# QLCH-ET-2017-06), collected samples from January 2017 to August 2017. Written informed consent and questionnaires were obtained from all subjects (children and legal guardians) visiting the outpatient gastroenterology unit at the Qilu Children's Hospital. They agreed to serve as fecal donors, in compliance with national legislation and the Code of Ethical Principles for Medical Research Involving Human Subjects of the World Medical Association (Declaration of Helsinki). For this study, children with symptoms of dyspepsia, aged 4 to 14 years, were recruited. All children were from the same geographical area. All symptomatic cases underwent ^13^C-urease breath test, abdominal ultrasonography, rapid urease test (RUT), endoscopy, and histopathological examination of gastric biopsy samples. Patients with gastritis indicating positive histopathology and RUT were diagnosed with *Helicobacter pylori* infection for the first time and were then divided into HPG, whereas those with negative histopathology and RUT results were divided into the HNG (Koletzko et al., 2011; Jones et al., 2017). When one of the histopathology and RUT results is negative, it can be combined with other test results, such as ^13^C-urease breath test. The exclusion criteria screened out patients with duodenal or gastric ulcers; active gastrointestinal bleeding; previous eradiation failure; or history of gastric surgery or drug therapy with antibiotics, probiotics, or gastric acid-suppressing drugs in the preceding 4 weeks. Also, healthy children (volunteers) from the same geographical area with matching age and sex composition as the other groups constituted the control group. They were all healthy with no known family history of digestive disease and medication for gastric disorders. Their ^13^C-urease breath test results were negative (Figure 1). Data were collected by using a standardized questionnaire including basic information, medical history, family history, and examination results.

### Sample Collection, DNA Extraction, and Sequencing

Fecal specimens were collected with sterilized 2-ml tubes containing pure ethanol on ice, immediately frozen (within 30 min), and stored at −80°C until analysis. Genomic DNA was extracted using cetyl trimethylammonium bromide (CTAB) method (Wang X. et al., 2018). An equivalent of 1 μl of each sample was used for DNA quantification using NanoDrop 2000 (Thermo Scientific). To analyze the bacterial population and amplification of the variable region, V1–V2 of the 16S rRNA gene was performed. PCR was conducted using bacterial universal primers 27F (5′AGAGTTTGATCMTGGCTCAG3′) 355R (5′GCTGCCTCCCG TAGGAGT 3′). The PCR products were checked using electrophoresis in 1% (w/v) agarose gels in TBE buffer (Tris, boric acid, and EDTA) stained with Genecolour I™ (Gene-bio) and visualized under UV light. Amplicons were first purified using the QIA quick PCR Purification Kit (Qiagen, Barcelona, Spain), quantified using a NanoDrop 2000 (Thermo Scientific), and then pooled in equal concentration. Pooled amplicons (2 nM) were then subjected to sequencing, using Illumina HiSeq 2500, following standard Illumina platform protocols.

### Analysis of 16S rRNA Gene Sequence

The 16S rRNA gene sequence paired-end data set was joined and quality filtered using the FLASH method. All sequence analyses were conducted in the Quantitative Insights Into Microbial Ecology (QIIME, version 1.9.1) software suite (Caporaso et al., 2010), as per the QIIME tutorial (http://qiime.org/). Chimeric sequences were removed using usearch61 with *de novo* models. Sequences were clustered against the 2013 Green genes (13_8 release) ribosomal database, 97% reference data set. Sequences that did not match any entries in this reference were subsequently clustered into *de novo* operational taxonomic units (OTUs) at 97% similarity with UCLUST. Taxonomy was assigned to all OTUs using the RDP classifier within QIIME and the Greengenes reference data set (Cole et al., 2009).

### Statistical Analysis

The questionnaires were analyzed using SPSS version 15.0 (SPSS Inc, Illinois, USA). There was no significant difference in age, sex, and indication for endoscopy between the groups (Table 1). To account for any bias caused by uneven sequencing depth, the least number of sequences present in any given sample was selected randomly from a sample category, prior to calculating community-wide dissimilarity measures (alpha diversity and beta diversity). The OTU table was then rarified to a sequencing depth of 22,000 per sample, for both diversity analyses. All principal coordinate analyses (PCoAs) were based on unweighted and weighted UniFrac distances, using evenly sampled OTU abundances. Linear discriminant effect size (LEfSe) analysis was performed to find features (taxa) differentially represented between patients and healthy controls. LEfSe combines Kruskal–Wallis test or pairwise Wilcoxon rank-sum test with linear discriminant analysis (LDA). It ranks features by effective size, which explains most of the biological differences at the top. LEfSe analysis was performed on the basis of the threshold of logarithmic LDA score for discriminative features, which is equal to 2.0. The effects of each of the two factors, that is, age and gender on the validated biomarkers, were examined within each of the two clinical categories by SPSS, in one-way ANOVA test. The effects were considered significant if *P-*value was < 0.05. The prediction of the functional composition of a metagenome, using marker gene data and a database of reference genomes, was done with Phylogenetic Investigation of Communities by Reconstruction of Unobserved States (PICRUSt) (Langille et al., 2013). The graphical representation of the results was done with R (McMurdie and Holmes, 2013) and STAMP. The calculation of *P-*values was done with Kruskal–Wallis *H*-test and Welch's *t*-test. Differences were considered significant when *P* < 0.05.

**Table 1:** Demographic and clinical characteristics of the patients.

**Group**	**HPG**	**HNG**	**HCG**	***P*-value**
	**(*n* = 50)**	**(*n* = 42)**	**(*n* = 62)**	
Mean age (±SD)	8.27 ± 2.8	8.64 ± 2.35	8.32 ± 1.21	0.5, 0.9, 0.42
**Gender %**				
Female	22 (44)	12 (28.6)	26 (41.9)	0.263
Male	28 (56)	30 (71.4)	36 (58.1)	
**Indication for endoscopy (%)**				
Recurrent abdominal pain	38 (76)	40 (95.2)		0.011
Nausea and (or) vomiting	31 (62)	23 (54.8)		0.482
Acid reflux symptoms	12 (24)	6 (14.3)		0.242
Anorexia	12 (24)	13 (30.9)		0.455
Abdominal swelling	9 (18)	9 (21.4)		0.68
Others	2 (4)	3 (7.1)		0.508

*The first P-value represents the following comparisons: HPG vs. HNG; HPG vs. HCG; and HNG vs. HCG*.

*HPG, Helicobacter pylori-induced gastritis group; HNG, H. pylori-negative gastritis group; HCG, healthy control group*.

## Results

### Demographic and Clinical Characteristics of Subjects

210 children with dyspeptic symptoms and 64 matched healthy children were initially screened for the study. One hundred individuals refused to participate, and 19 children were ruled out, and one fecal specimen was missing. Around 154 individuals were involved (Figure 1 and Table 1), comprising 50 children in HPG (mean age at 8.27 ± 2.8 years, 22 girls and 28 boys). Forty-two children with gastritis, excluding *Helicobacter pylori* infection, were included in HNG (mean age at 8.64 ± 2.35 years, 12 girls and 30 boys), whereas 62 healthy children (mean age at 8.41 ± 1.17 years, 26 girls and 36 boys) were included in HCG. Fecal specimen and questionnaires were collected from all subjects.

### The Diversity of Gut Microbiome in Three Groups of Children

**Figure 2:**
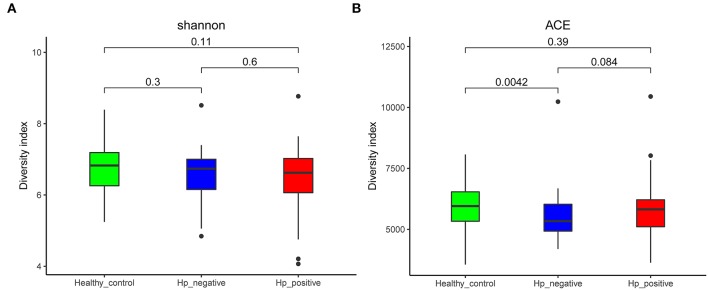
Comparison of alpha diversity (**A**, Shannon index; and **B**, ACE index) based on the OTU profile. HPG, HNG, and HCG are colored in red, blue, and green, respectively. The *P*-value was calculated by the Wilcoxon rank-sum test. HPG, *Helicobacter pylori*-induced gastritis group; HNG, *H. pylori*-negative gastritis group; HCG, healthy control group; OUT, operational taxonomic unit.

We compared the richness (abundance-based coverage estimator [ACE]) and diversity (Shannon) of bacterial community among HNG, HPG, and HCG (Figure 2). There were no significant differences in Shannon and ACE index in the comparison of three groups, except for ACE index in comparing HNG and HCG (*P* = 0.0042, Figure 2A).

**Figure 3:**
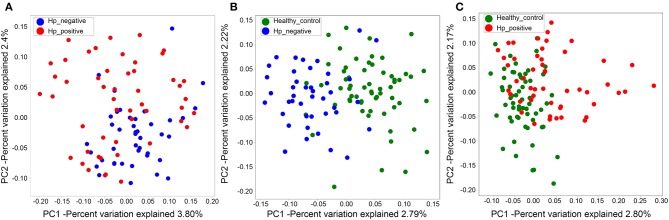
PCoA of bacterial beta diversity based on the unweighted UniFrac distance. **(A)** Between HPG and HNG. **(B)** Between HNG and HCG. **(C)** Between HPG and HCG. PCoA, principal coordinate analysis; HPG, *Helicobacter pylori*-induced gastritis group; HNG, *H. pylori*-negative gastritis group; HCG, healthy control group.

We also evaluated beta diversity among the three groups using PCoA, on the basis of the unweighted UniFrac distances. The PCoA demonstrated clustering of microbial communities between HNG and HPG (Figure 3A), HCG and HNG (Figure 3B), and HCG and HPG (Figure 3C). We used analysis of similarities (ANOSIM) to test whether two groups are significantly different in PCoA. Results indicated that there was a significant difference in gut microbiome structure between HNG and HPG (*P* = 0.002, *R* = 0.055, ANOSIM), HCG and HNG (*P* = 0.001, *R* = 0.178, ANOSIM), and HCG and HPG (*P* = 0.001, *R* = 0.187, ANOSIM).

### Composition of Gut Microbiome of Children in All Three Groups

**Figure 4:**
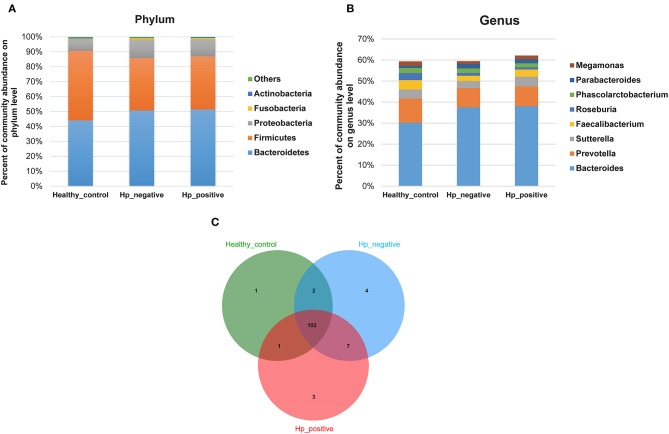
Comparison of relative taxa abundance between HPG, HNG, and HCG. **(A)** Comparison of relative taxa abundance among HPG, HNG, and HCG at the phylum level. **(B)** Comparison of relative taxa abundance among HPG, HNG, and HCG at the genus level. **(C)** Venn diagram. HPG, *Helicobacter pylori*-induced gastritis group; HNG, *H. pylori*-negative gastritis group; HCG, healthy control group.

In the relative taxon abundance of groups, using 97% as the similarity cutoff, 605 qualified taxa were identified. At the phylum level, the gut microbiome profiles in the three groups of subjects were dominated by Bacteroidetes (HPG 51.37 ± 16.15%, HNG 50.71 ± 18.34%, and HCG 44.07 ± 14.68%), Firmicutes (HPG 35.65 ± 11.92%, HNG 35.16 ± 14%, and HCG 46.54 ± 13.78%), Proteobacteria (HPG 11.53 ± 12.13%, HNG 12.32 ± 9.49%, and HCG 8.09 ± 5.62%) (Figure 4A) (Caporaso et al., 2010; Langille et al., 2013; McMurdie and Holmes, 2013). Phylum level analysis also demonstrated the ratios of Firmicutes/Bacteroidetes (F:B), which had dramatically decreased in HPG (*P* = 0.012) and HNG (*P* = 0.0039), as compared with HCG. However, there was no difference between HPG and HNG in F:B ratio, implying that the bacterial community in fecal microbiome may alter in the disease state. At the genus level, *Bacteroides* (HPG 38.03 ± 17.47%, HNG 37.47 ± 17.81%, and HCG 30.18 ± 15.26%) and *Prevotella* (HPG 9.41 ± 14.96%, HNG 9.17 ± 15.1%, and HCG 11.45 ± 16.58%) were the major genus. There was also increased abundance of *Bacteroides* and *Parabacteroides*, decreased abundance of *Roseburia* and *Faecalibacterium* in HPG and HNG, compared with HCG (Figure 4B). Additionally, the Venn diagram shows 104 common OUTs of bacteria among the three groups (Figure 4C). There are four, three, and one unique OTUs for HNG, HPG, and HCG, respectively.

### Differential Taxonomic Abundance in Three Groups of Children

**Figure 5:**
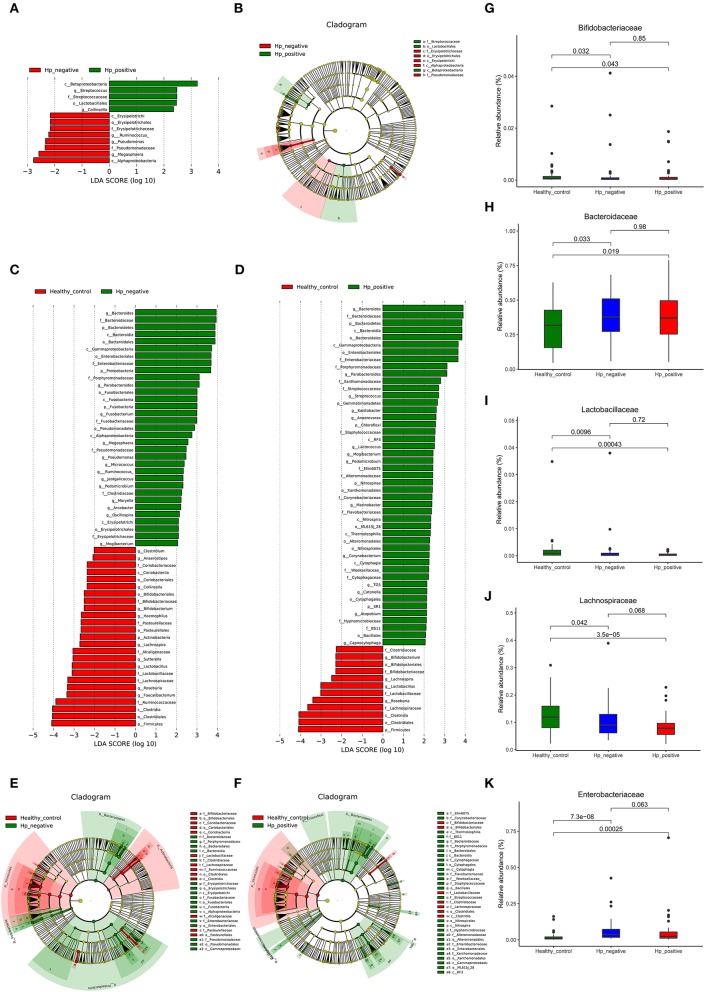
Characteristics of microbial community composition in HPG, HNG, and HCG. **(A)** The most differentially abundant taxa between HPG and HNG (LDA score above 2), which was generated from LEfSe analysis. **(B)** The enriched taxa of fecal microbiome in HPG and HNG are represented in the cladogram. The central point represents the root of the tree (bacteria), and each ring represents the next lower taxonomic level (phylum to genus: p, phylum; c, class; o, order; f, family; g, genus). **(C)** The most differentially abundant taxa between HNG and HCG (LDA score above 2), which was generated from LEfSe analysis. **(D)** The most differentially abundant taxa between HPG and HCG (LDA score above 2), which was generated from LEfSe analysis. **(E)** Enriched taxa of fecal microbiome in HNG and HCG are represented in cladogram. The central point represents the root of the tree (bacteria), and each ring represents the next lower taxonomic level (phylum to genus: p, phylum; c, class; o, order; f, family; g, genus). **(F)** The enriched taxa of fecal microbiome in HPG and HCG are represented in cladogram. The central point represents the root of the tree (bacteria), and each ring represents the next lower taxonomic level (phylum to genus: p, phylum; c, class; o, order; f, family; g, genus). **(G–K)** Relative abundances of five bacteria (Bacteroidaceae, Enterobacteriaceae, Bifidobacteriaceae, Lactobacillaceae, and Lachnospiraceae) among HPG, HNG, and HCG were compared. HPG, *Helicobacter pylori*-induced gastritis group; HNG, *H. pylori*-negative gastritis group; HCG, healthy control group; LDA, linear discriminant analysis; LEfSe, linear discriminant effect size.

We applied LEfSe analysis to further identify the significantly different abundance between HNG and HPG, HCG and HNG, and HCG and HPG. Results revealed 13 taxa, distinguishing the gut microbiome communities with HNG and HPG, using an LDA score of above 2 (Figure 5A). There were 59 taxa each, distinguishing the gut microbiome communities with HCG and HNG (Figure 5C) and HCG and HPG (Figure 5D), respectively. A cladogram (Figures 5B,E,F) was used to represent predominant bacteria and the structure of the microbiota in each group. To assess the impact of *H. pylori* on the gut microbiome in children, the fecal microbiome in HPG and HNG was analyzed (Figure 5A). There was a higher abundance of Betaproteobacteria and Lactobacillales and lower abundance of Alphaproteobacteria in HPG. At the family and genus levels, higher abundance of *Streptococcus* and *Collinsella* and lower abundance of Pseudomonadaceae, Erysipelotrichaceae, and *Megasphaera* were found in HPG, as compared with HNG. Additionally, the compositions of gut microbiome between HNG and HCG were compared to analyze the altered gut microbiome in gastritis. An abundance of Bacteroidaceae, Enterobacteriaceae, Porphyromonadaceae, Fusobacteriaceae, and *Megasphaera* was seen in HNG. However, a higher abundance of Ruminococcaceae, Lachnospiraceae, Bifidobacteriaceae, *Roseburia, Lactobacillus, Sutterella*, and *Bifidobacterium* were found in the HCG (Figure 5C). The compositions of gut microbiome in children, HPG, and HCG were also compared and analyzed. Significant differences in taxa from phylum to genus level were represented in LDA. At the family and genus levels, Bacteroidaceae, Enterobacteriaceae, Porphyromonadaceae, *Bacteroides, Parabacteroides, Streptococcus*, and *Lactococcus* numbers increased significantly in HPG (Figure 5D). Lastly, the compositions of gut microbiome in HNG and HCG, and HPG and HCG were compared at five significant bacterial levels. There was significantly higher abundance of Bacteroidaceae and Enterobacteriaceae and lower abundance of *Bifidobacteriaceae, Lactobacillaceae*, and *Lachnospiraceae* in HPG and HNG, compared with HCG (Figures 5G–K). Thus, these results imply that gastric inflammation significantly changes the composition of gut microbiome, especially in children with *H. pylori-*induced gastritis.

### Predictive Function of Gut Microbiome in Three Groups of Children

**Figure 6:**
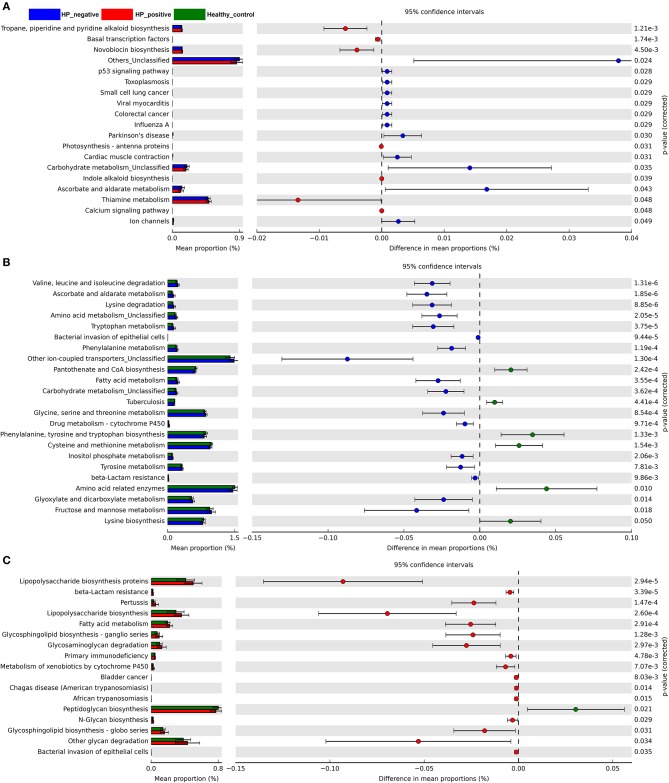
Predicted metagenome function based on KEGG pathway analysis. Extended error bar plots show the significantly different abundance of KEGG pathways. **(A)** Between HPG and HNG. **(B)** Between HNG and HCG. **(C)** Between HPG and HCG. The proportion (left side) indicates the possible abundance of microbes possessing each functional feature and the difference between proportions for each feature. Circles (right side) represent the difference between the mean proportion of bacteria (the effect size), adjacent to their respective CI (error bars). KEGG, Kyoto Encyclopedia of Genes and Genomes; HPG, *Helicobacter pylori*-induced gastritis group; HNG, *H. pylori*-negative gastritis group; HCG, healthy control group.

We also explored microbiota function on the basis of inferred metagenomes using the PICRUSt algorithm. We compared the differences between HNG and HPG, HCG and HNG, and HCG and HPG in the Kyoto Encyclopedia of Genes and Genomes (KEGG) orthology (KO). Among the 328 affiliated KEGG pathways, 19 had statistically significant and different *P* < 0.05 in HNG and HPG (Figure 6A). Interestingly, pathways related to genetic information (basal transcription factors) and environmental information processing (calcium signaling pathway) were enriched in HPG. Pathways related to human diseases (toxoplasmosis, small-cell lung cancer, and colorectal cancer), cellular processes (p53 signaling pathway), and metabolism (carbohydrate metabolism) were depleted in HPG. And there were 23 KEGG pathways showing significant differences in HCG and HNG (Figure 6B). Pathways related to metabolism (amino acid, fatty acid, carbohydrate, and drug metabolism—cytochrome P450 and beta-lactam resistance) and human disease (bacterial invasion of epithelial cells) were enriched in HNG. However, cellular processes, signaling (other ion-coupled transporters) and other metabolism (pantothenate and CoA biosynthesis, phenylalanine, tyrosine and tryptophan biosynthesis, and lysine biosynthesis) were depleted in HNG. Seventeen KEGG pathways showed significant differences in HCG and HPG (Figure 6C). Pathways related to metabolism (lipopolysaccharide [LPS] biosynthesis proteins, beta-lactam resistance, LPS biosynthesis, fatty acid metabolism, glycosphingolipid biosynthesis–ganglio series, glycosaminoglycan degradation, metabolism of xenobiotics by cytochrome P450, N-glycan biosynthesis, glycosphingolipid biosynthesis–globo series, and other glycan degradation) and human disease (pertussis, primary immunodeficiency, bladder cancer, Chagas disease, African trypanosomiasis, and bacterial invasion of epithelial cells) were enriched in HPG, but peptidoglycan biosynthesis was found depleted in HPG. These findings show that gut microbiome affected by *H. pylori* infection and gastritis also causes some changes in the body's basal metabolic function, disease susceptibility, and drug metabolism. It can also be said that gut microbiome influences gastric inflammation. These results thus indicate that in children, *H. pylori* infection, gastritis, and altered intestinal microbiome interact with each other.

## Discussions

Microbiome dysbiosis has been linked to gastrointestinal disease including gastritis, in which *Helicobacter pylori* plays an important role (He et al., 2016; Minalyan et al., 2017; Sgambato et al., 2017; Gorkiewicz and Moschen, 2018). Although there are several studies addressing bacterial biodiversity in upper GI tract, the role of *H. pylori* infection and gastritis in the gut bacterial community, especially in children, is unknown. A preliminary study evaluated the influence of *H. pylori* infection and gastritis on fecal microbiome by comparing three pediatric groups, using 16S rRNA gene sequence analysis. This study revealed (i) significant differences in beta diversity analysis in the three groups, especially in HPG, HNG, and HCG; (ii) F:B ratio dramatically decreased in both HPG and HNG, with higher abundance of Bacteroidaceae and Enterobacteriaceae and lower abundance of Lachnospiraceae, Bifidobacteriaceae, and Lactobacillaceae also found in HPG and HNG; and (iii) HPG had higher abundance of Betaproteobacteria, Lactobacillales and *Streptococcus*, lower abundance of Alphaproteobacteria, *Megasphaera*, than HNG. The results indicate that *H. pylori* infection and gastritis could alter gut microbiome.

Unlike adults, *H. pylori*-infected children were mostly asymptomatic with different microscopic gastric inflammation, and only a small proportion developed clinical manifestations of chronic infection, for example, peptic ulcer and gastric cancer (Jones et al., 2017). With declining prevalence in children, the incidence of early-onset asthma, inflammatory bowel disease (IBD), gastrointestinal and systemic infections, and Barrett's esophagus increased (Arnold et al., 2011; Cohen et al., 2012; Castano-Rodriguez et al., 2017; Minalyan et al., 2017). These were explained by the tolerogenic immune-state induced by *H. pylori* at an early age, which helps bacteria persist in the human host (Gorkiewicz and Moschen, 2018). Hence, *H. pylori* has been considered as a late-in-life human pathogen with potential early-life benefits. The decrease in eradication rate and increase in drug resistance are the main problems in children. There is no clear consensus regarding the optimal age for *H. pylori* eradication therapy (Gotoda et al., 2018). Moreover, the relationship between *H. pylori*, IBD and colon cancer is currently unclear. Investigating fecal microbiome changes induced by *H. pylori* infection and gastritis could be beneficial in the assessment of consequences, perpetrated by *H. pylori* infection in children and also in exploring new treatment strategies.

Although the exact mechanism of how gastric inflammation leads to changes in fecal microbiota are not clearly defined, there is increasing evidence that interplay between bacteria and host responses may shape commensal microbiota composition. Changes in the gastric microbiota, luminal pH, and end products of bacterial fermentation play important roles in driving the community structure of gut microbiota (Cremer et al., 2017). However, the relationship between gastritis and gut microbiome has been underreported (Chen et al., 2018; Gao et al., 2018). Juan-Juan Gao et al. analyzed fecal microbiome in patients with different *H. pylori*-related gastric lesions. They found alterations in dominant phyla of Bacteroidetes, Firmicutes, and Proteobacteria in groups with different *H. pylori* status (Gao et al., 2018). These had never been observed in related studies in children. The results of the current study first showed that different floras in HNG and HCG before treatment were impacted by simple gastritis. Our data suggested that the abundance of Bacteroidaceae, Enterobacteriaceae, Porphyromonadaceae, Fusobacteriaceae, *Bacteroides*, and *Megasphaera* increased in the HNG. Interestingly, most of the significant taxa belonged to Gram-negative bacteria producing LPS. The LPS from intestinal microbiome induces a chronic subclinical inflammatory process (Saad et al., 2016). These results together indicate that variation in fecal microbiome could be an additional risk factor promoting gastrointestinal inflammation in children.

*H. pylori* infection is the main cause of gastritis and has developed mechanisms to coexist in the harsh gastric microenvironment, where it induces mucosal inflammation, immune activation, hypergastrinemia, and variable effects on gastric acid production. On the basis of our findings, we concluded that HPG had a higher abundance of Lactobacillales and *Streptococcus*. In our study, the abundance of Lactobacillaceae and *Lactobacillus* all decreased in HPG and HNG, but in HPG, there was higher abundance of *Lactobacillales*. *Lactobacillales* belong to Bacilli class and Firmicutes phylum and have six families. Lactobacillaceae and *Lactobacillus* did not increase or decrease when HPG and HNG were compared. However, *Streptococcus* genus, which belongs to the Streptococcaceae family, increased significantly in HPG. Hence, we correlated the rich abundance of Lactobacillales with higher abundance of *Streptococcus* genus. *Streptococcus* belongs to commensals like oral microbiome (Nasidze et al., 2009) and healthy esophageal core microbiome (Hunt and Yaghoobi, 2017; Nardone et al., 2017). Khosravi et al. reported that *Streptococcus mitis* induced the conversion of *H. pylori* to coccoid cells in coculture studies and inhibited its growth (Khosravi et al., 2014a). Furthermore, a study found that (insulin–gastrin) INS-GAS mice coinfected with *H. pylori* and *Streptococcus salivarius* developed more severe gastric inflammation than did *H. pylori* only at 5 months post-infection (Rizzato et al., 2019). *Streptococcus* has also been associated with peptic ulcer (Khosravi et al., 2014b) and gastric carcinogenesis (Coker et al., 2018). Hence, *Streptococcus* genus exists in normal gastrointestinal tract and interacts with gastric diseases. Our study also concluded that a lower abundance of *Megasphaera* had been found in HPG, compared with HNG. *Megasphaera*, a genus of *Firmicutes* bacteria within the class Negativicutes, has been found in the stomach of cattle and sheep and in human fetuses. A type of *Megasphaera* species, named *Megasphaera elsdenii*, inhabits the human intestine and has probiotic potential (Kwon et al., 2018). Owing to technological limitations, the real bacterial response in humans still needs to be confirmed in further studies.

In conclusion, fecal microbiome was affected by *H. pylori* in patients with gastritis. However, comparisons of compositions of gut microbiome in HPG and HCG also confirmed the above alteration in fecal microbiome in gastritis and *H. pylori* infection. At the same time, it also shows that most changes in intestinal flora are caused by gastric infection. However, factors caused by *H. pylori* infection can also cause alterations in the quantum of some special bacteria. *Streptococcus* and *Megasphaera* were found in abundance in HPG and HNG. This is consistent with symptoms of indigestion observed in patients with *H. pylori*-induced gastritis and common gastritis (Correa Silva et al., 2016; Jones et al., 2017).

With respect to the altered fecal microbiome composition in gastritis, the F:B ratio dramatically decreased in HPG and HNG. The reduced ratio of F:B in *H. pylori*-positive subjects before treatment has also been found in another study (Osaki et al., 2018). It is well-known that the ratio of F:B is related to obesity, body mass index (BMI), metabolic syndrome, elderly people, and the eradication of *H. pylori* infection (Azuma et al., 2002; Claesson et al., 2011; Takeoka et al., 2016). The current study did not measure the BMI of all individuals; further research is needed to verify the interaction between the ratio of F:B and *H. pylori* infection. Higher abundance of Enterobacteriaceae and *Bacteroides* was also found in groups. There are many kinds of pathogenic bacteria in Enterobacteriaceae, like *Escherichia, Salmonella*, and *Shigella*. Enterotoxigenic *Escherichia coli* (ETEC) can produce enterotoxin. It is known that ETEC increases the release of diamine oxidase (DAO) and d-lactate in the plasma, which can lead to damage of the intestinal epithelial cell membrane (Liu et al., 2017). The up-regulation of pro-inflammatory cytokines and down-regulation of anti-inflammatory cytokines (Xun et al., 2015) may be a way to influence gastritis. *Bacteroides fragilis*, which accounts for only 0.5% of the human colonic microbiome, is the most commonly isolated anaerobic pathogen. In addition, a number of *Bacteroides* spp. have high resistance to antibiotics (Mazmanian et al., 2005; Wexler, 2007). Several studies have reported an increase or decrease in Bacteroidales in mucosal samples of IBD subjects compared with controls (Zitomersky et al., 2013), which indicates that Bacteroidales are connected with IBD. Abundance of two taxa (Enterobacteriaceae and *Bacteroides*) is connected with intestinal inflammation and IBD.

Generally, two genera of *Lactobacillus* and *Bifidobacterium* are considered as probiotics, which are important to human health (Wang Y. et al., 2018). This study revealed that the two genera significantly decreased in the gut microbiome of children in HPG and HNG. They can change the PH of the intestinal environment to inhibit the growth of pathogenic bacteria and stimulate an immune response (Zhu et al., 2018). Some studies also concluded that *Lactobacillus* spp. in the gastric microbiome also has an inhibitory effect against *H. pylori* (Zaman et al., 2014; Salas-Jara et al., 2016). In another study, children with *H. pylori* infection had a decreased relative abundance of *Bifidobacterium*, which significantly increased after the children ingested probiotics-containing yogurt (Yang and Sheu, 2012). Higher abundance of *Lactobacillus* and *Bifidobacterium* in healthy children could protect them from gastrointestinal inflammation. But some other studies also have opposite results from *Lactobacillus*. A recent study on *Lactobacillus* and *H. pylori* coisolates from humans did not reveal any significant influence of lactobacilli on *H. pylori* strains (Skoog et al., 2011). Iino et al. also found that *H. pylori* infection initially influenced the composition ratio of each *Lactobacillus* species in the gut microbiota before atrophic gastritis progression and suggested a higher abundance of *Lactobacillus* in *H. pylori*-infected subjects with severe atrophic gastritis (Iino et al., 2018). A German study also reported that *H. pylori* led to an increased growth of lactobacilli in fecal microbiome (Buhling et al., 2001). These results were considered to take into account long-term acid suppression induced by *H. pylori* infection and PPI therapy following atrophic gastritis (Takashima et al., 2001; Weck et al., 2009; Jackson et al., 2016). This suggests that lactic acid-producing bacteria may also enhance gastric inflammatory reactions caused by *H. pylori*. There is still controversy in the species and dosage of *Lactobacillus* as probiotic with *H. pylori* infection (Schulz et al., 2015; Iino et al., 2018). Lower abundance of two genera in the gut microbiome of children in HPG may promote the production of inflammatory factors, leading to gastritis. However, before the results of our study are used for the treatment of *H. pylori-*induced gastritis with *Lactobacillus* and *Bifidobacterium*, the two bacterial species need to be confirmed in future studies.

To compare the detailed altered KEGG pathway, significant differences in colorectal cancer occurrence between the two groups have been associated with specific changes in gut microbiome composition. A metagenome-wide association study (MGWAS) was performed, and it was found that certain *Bacteroides* spp. (e.g., *Bacteroides dorei, Bacteroides vulgatus*, and *Bacteroides massilensis*) and *E. coli* were associated with systemic inflammation and tumor staging (Feng et al., 2015). Since the last two decades, several studies investigated the potential association of *H. pylori* infection with colorectal neoplasia (Breuer-Katschinski et al., 1999; Mizuno et al., 2005; Inoue et al., 2011; Papastergiou et al., 2016). However, direct activation of colorectal carcinogenesis by the bacterium remains controversial (Papastergiou et al., 2016). Our data suggest that higher abundance of Bacteroidaceae and *Enterobacteriaceae* was also found in the HNG and HPG, and the KEGG pathway of colorectal cancer increased in HNG, similar to another study (Chen et al., 2018), whereas most studies have reported that colorectal cancer pathways were predicted to be higher in the *H. pylori*-positive group (Kountouras et al., 2018; Kumar et al., 2018). It is necessary to confirm the true relationship of *H. pylori*, gastritis, and colorectal cancer by further research on gut microbiome alteration.

Our study showed significant increase of activity in metabolic pathways of children with HPG and HNG. This included fatty acid metabolism and beta-lactam resistance to drug due to cytochrome P450. *H. pylori* depends on unsaturated fatty acid (UFA) biosynthesis for maintaining its membrane structure and function (Bi et al., 2016). The level of microbial UFA is significantly elevated in the blood of patients with *H. pylori* infection-induced peptic ulceration (Ktsoyan et al., 2010). These results suggest that *H. pylori* infection is related to higher lipid metabolism. The functional analysis of microbiome revealed that lipid metabolism pathway increased in the group with gastritis, indicating that gut microbiome has similar effects as *H. pylori*-induced gastritis. *H. pylori* eradication is affected by antibiotic resistance and genotypes (Chunlertlith et al., 2017). Increased antibiotic resistance, such as beta-lactam and cytochrome P450 CYP2C19 gene expression that could encode a protein degrading PPI decreases the eradication rate of *H. pylori* (Boyanova et al., 2016). Results showed that beta-lactam resistance and cytochrome P450 pathways increased in children of both HPG and HNG, which is important information for developing alternative or improved treatment strategies. Thus, the body status of the glucose and lipid metabolism, beta-lactam resistance, metabolism of xenobiotics by cytochrome P450 and drugs by cytochrome P450 may be connected to gastritis. Treatment effectiveness of *H. pylori*-induced gastritis in children can be increased by altering fecal microbiome, especially the eradication rate of *H. pylori*.

Widespread use of antibiotics in children for *H. pylori* gastritis decreases efficacy of *H. pylori* eradication therapy and increases prevalence of antibiotic-resistant strains. This study reports the difference in fecal microbiome observed in infection caused by *H. pylori* and gastritis. *Streptococcus* and *Megasphaera* were found in gut microbiome in HPG and HNG. The altered categories within the KEGG pathway suggest that these different bacteria may play a role in the drug resistance of *H. pylori* infection or the relationship with colon cancer and IBD. These results suggest that gastritis itself can cause changes in composition of fecal microbiome, which may be exacerbated by *H. pylori* infection. These changes in gut microbiome may be related to drug resistance and the development of chronic gastrointestinal diseases, affecting the treatment of *H. pylori* infection and gastritis. *Lactobacillus* and *Bifidobacterium* can be used as probiotics to treat gastritis patients. This indeed suggests the close interaction of *H. pylori* infection, gastritis, and gut microbioma. Hence, the decision to investigate and treat the infection should be assessed by a clear benefit for the individual child. Reasonable and effective treatment should be selected for children to avoid repeated antibiotic use for intestinal flora. The use of probiotics is expected to be used to treat children with mild illness or to alleviate symptoms. But the efficacy, species, and dosage of probiotics, however, need to be further studied.

In conclusion, this study first demonstrated the structural, compositional, and functional dysbiosis of fecal microbiome in gastritis caused by *H. pylori*. It indicated that the current treatments combined with strategies that modulate the gut microbiome could improve the clinical outcome of *H. pylori-*induced gastritis. The findings may pave the way for initiating larger-cohort clinical validations and developing guidance for therapeutic strategies with probiotics. However, the study also has some shortcomings; for example, the technology is accurate for only a few species, and the corresponding specimen of gastric mucosa and blood samples of individuals were not collected to compare with the same fecal specimen. In addition, the number of involved children was less. In future, a larger study is required, and detailed clinical data need to be collected to confirm these results.

## Data Availability Statement

All sequencing data associated with this study were uploaded to the NCBI SRA database (accession number: PRJNA544571). The webpage of the SRA database is https://www.ncbi.nlm.nih.gov/sra.

## Ethics Statement

The studies involving human participants were reviewed and approved by Medical Ethics Committee of Qilu Children's Hospital of Shandong University. Written informed consent to participate in this study was provided by the participants' legal guardian/next of kin.

## Author Contributions

LZ and ZG designed the study. LY, JZ, CZ, and JY performed the measurements and data analysis. JX, XW, YL, HL, and YW obtained the samples and clinical details. LY and JZ wrote the manuscript. All authors have read and critically revised the manuscript.

### Conflict of Interest

The authors declare that the research was conducted in the absence of any commercial or financial relationships that could be construed as a potential conflict of interest.
